# Pseudovasculitic Scurvy Mimicking Small Vessel Vasculitis: A Case Report

**DOI:** 10.3390/reports9020114

**Published:** 2026-04-08

**Authors:** Andrea C. R. Chieng, Branavan Sivagnanam, Magnus H. Liew, Priyani Daluwatte

**Affiliations:** Department of Internal Medicine, Goulburn Valley Health, Shepparton, VIC 3630, Australia; brana.siva@gmail.com (B.S.); bingbin100@gmail.com (M.H.L.); priyaniadaluwatte@gmail.com (P.D.)

**Keywords:** scurvy, vitamin C deficiency, pseudovasculitis, small vessel vasculitis, petechiae, malnutrition, restricted diet

## Abstract

**Background and clinical significance**: Scurvy, resulting from vitamin C deficiency, is rare in the 21st century, particularly in high-income settings. Its nonspecific presentation and ability to mimic a wide range of conditions make diagnosis incredibly challenging. Pseudovasculitic petechial lesions of the lower limbs may be misdiagnosed as systemic vasculitis, often leading to extensive investigations and delayed treatment. **Case presentation**: We report the case of a 45-year-old woman who presented with progressive lower limb pain, swelling, and vasculitis-like petechial rash with ecchymoses. Extensive investigations for autoimmune, infectious, malignant, and vascular causes, including skin biopsy, were unremarkable. A detailed dietary history revealed markedly restrictive intake. Characteristic dermatological findings, including perifollicular haemorrhage, ecchymoses and hair shaft abnormalities, raised suspicion for scurvy. Profoundly reduced serum ascorbic acid levels confirmed vitamin C deficiency. **Conclusions**: This case highlights the importance of thorough dietary assessment, recognition of characteristic cutaneous features, and identification of risk factors such as restrictive eating patterns or chronic gastrointestinal symptoms. It underscores the need for a high index of clinical suspicion for scurvy, even in contemporary high-income settings. Early diagnosis and vitamin C supplementation can result in rapid clinical improvement and prevent avoidable morbidity.

## 1. Introduction and Clinical Significance

Scurvy caused by vitamin C deficiency was historically described among sailors during long sea voyages [1]. Vitamin C plays a critical role in multiple physiological processes, particularly collagen synthesis, which is essential for wound healing and tissue integrity. Its antioxidant properties also contribute to immune function by protecting cells from oxidative stress. As humans are unable to synthesise vitamin C endogenously, it must be obtained through diet and is absorbed in the terminal ileum [2]. Malnutrition remains the most common cause of scurvy. The recommended daily intake in adults is 90–120 mg, with higher requirements in smokers [3]. Although there is no definitive serum ascorbic acid threshold for clinical manifestations, symptoms generally appear after 4–12 weeks of inadequate intake [4]. Here, we report a case of pseudovasculitic scurvy presenting with vasculitis-like cutaneous features and cytopenias in a high-income setting.

## 2. Case Presentation

A 45-year-old woman presented with multiple visits to the emergency department over four weeks due to worsening pain and swelling of her lower extremities, mainly the right ankle, which limited her mobility. Her arthralgia was preceded by a five-month history of generalised petechiae rash, primarily involving both lower limbs extending up to the thighs, and to a lesser extent to the arms. More recently, she developed ecchymoses over the lower extremities. She denied altered bowel habits, recent upper respiratory tract infection or urinary symptoms such as haematuria. Her past medical history included chronic iron deficiency anaemia, ischemic heart disease, chronic obstructive pulmonary disease, gastroesophageal reflux, anxiety, depression and a recent diagnosis of gastritis and duodenitis. She also reported chronic dysphagia and abdominal pain, which were attributed to gastroparesis and cannabis induced vomiting. Despite extensive investigations by her gastroenterologist, no definitive cause had been identified. There was an unsuccessful attempt with the gastric emptying study, thus the patient declined further investigations.

There was no personal or family history of autoimmune disease. At baseline, she was fully independent at home. She was a former intravenous drug user and reported ongoing cannabis use. A detailed dietary history revealed a highly restrictive intake consisting predominantly of iced coffee for approximately three years, secondary to postprandial vomiting.

**Figure 1:**
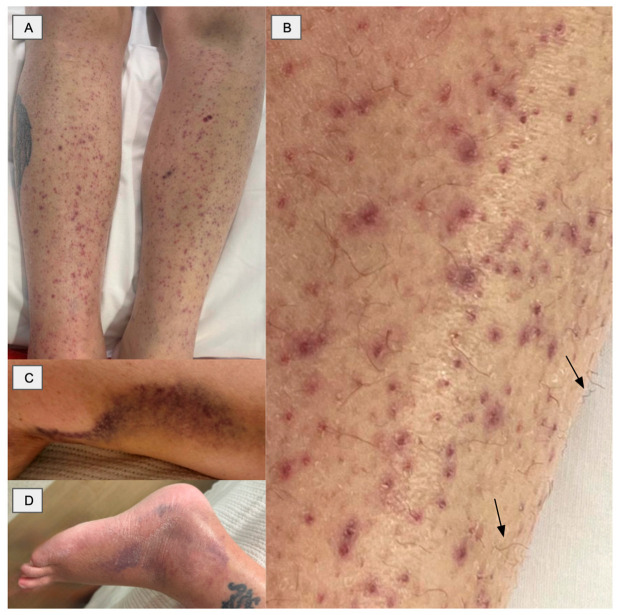
(**A**) Symmetrical perifollicular purpura of the lower limbs (**B**) Close-up showing perifollicular haemorrhage with subtle hair shaft abnormalities (arrows) suggestive of coiling (**C**) Confluent ecchymosis (**D**) Ankle ecchymosis with swelling.

Physical examination of the lower limbs showed perifollicular haemorrhage with petechiae, ecchymoses and subtle hair shaft abnormalities with mild coiling (Figure 1).

Laboratory findings (Table 1) demonstrated severe hypochromic microcytic anaemia, with mild leukopenia and thrombocytopenia. Peripheral blood film showed mild anisocytosis and hypochromia with mild polychromasia, without features suggestive of haematological malignancy. Iron studies including serum iron, ferritin and transferrin saturation were low. Mild electrolyte derangements were noted, including hyponatremia, hypokalemia and hypomagnesemia. Additional biochemical analysis showed low calcium and folate. Inflammatory markers, coagulation profile, renal and liver function tests were within normal limits.

Infectious disease, including hepatitis B and C serology, was negative. An extensive autoimmune workup, such as antinuclear antibodies, antineutrophil cytoplasmic antibodies, anti-double stranded DNA, antiphospholipid antibodies, rheumatoid factor, anti-cyclic citrullinated peptide, and extractable nuclear antigen panel, was negative. Complement levels (C3 and C4) were within normal limits. Further investigations, including immunoglobulins, cryoglobulins, and serum protein electrophoresis, were unremarkable. Thyroid function tests were normal. Evaluation for endocrinopathies, including Cushing syndrome, was unremarkable with normal cortisol and adrenocorticotropic hormone levels. Urinalysis showed no haematuria or proteinuria.

**Figure 2:**
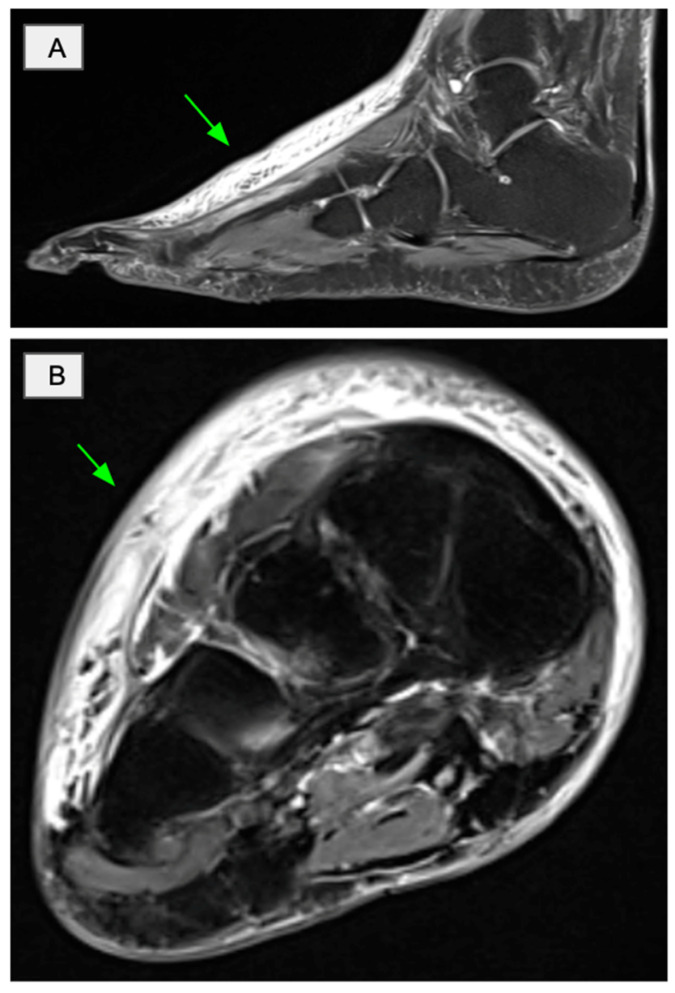
T2-weighted magnetic resonance imaging of the right ankle. (**A**) Sagittal and (**B**) axial views demonstrating hyperintense signal within the dorsal soft tissues (arrows), including the region of the flexor hallucis longus tendon sheath. These findings are consistent with non-specific soft tissue oedema, which may reflect underlying haemorrhage in the context of severe vitamin C deficiency.

Radiological investigations were largely unremarkable. Ultrasound of the lower limbs excluded deep venous thrombosis. Magnetic resonance imaging of right ankle and foot demonstrated nonspecific dorsal soft tissue swelling without abscess or collection, with a small amount of fluid along the flexor hallucis longus tendon sheath (Figure 2). No marrow oedema or other abnormalities were identified. Computed tomography of the chest, abdomen, and pelvis showed no evidence of malignancy.

Punch biopsy of the cutaneous lesions revealed features consistent with pigmented purpuric dermatoses with red blood cell extravasation and haemosiderin deposition, without evidence of vasculitis, leukocytoclasis, atypia or malignancy. Direct immunofluorescence for IgA, IgG, IgM, fibrinogen, C1q and C3 was negative.

**Figure 3:**
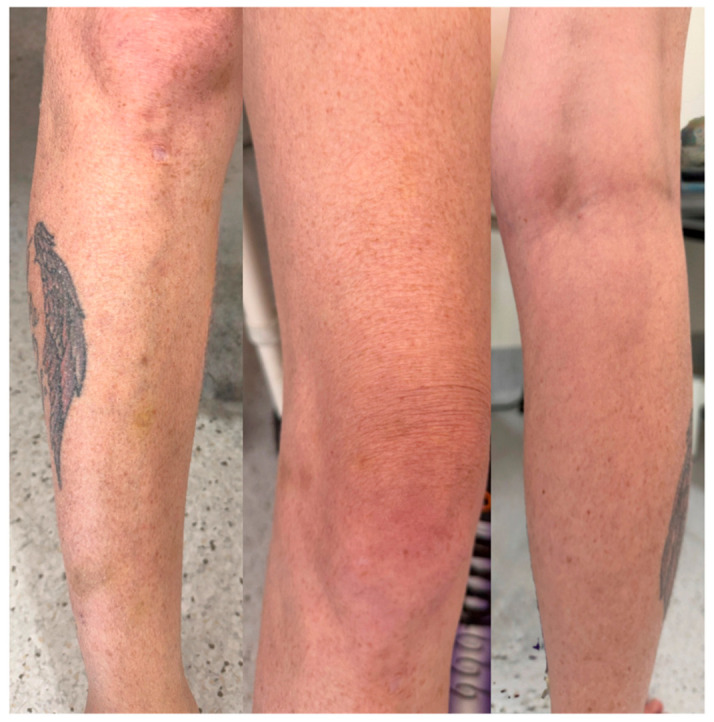
Clinical improvement within three months following vitamin C replacement, demonstrating resolution of perifollicular purpura and ecchymoses.

Given the characteristic perifollicular haemorrhage, hair shaft abnormalities, and markedly restrictive diet, nutritional deficiency was suspected. Serum ascorbic acid level was less than 3 μmol/L (reference range 23–85 μmol/L), confirming severe vitamin C deficiency. The patient was commenced on oral ascorbic acid at a dose of 1 g twice daily. At the three-month follow up, there was marked clinical improvement, with complete resolution of musculoskeletal symptoms, perifollicular haemorrhage and ecchymoses (Figure 3). Her gastrointestinal symptoms also improved with proton pump inhibitor therapy, allowing resumption of a regular diet.

## 3. Discussion

Scurvy is caused by vitamin C deficiency and remains a rare condition in modern clinical practice. However, it remains an important and often overlooked differential in individuals with significant nutritional deficiency. Recognised risk factors include alcohol use disorder, eating disorders, malabsorptive disorders, and prolonged dietary restriction [2]. In this case, the diagnosis was challenging due to the nonspecific nature of the presenting features, including progressive lower limb pain and swelling, preceded by petechiae and ecchymosis. The presence of perifollicular haemorrhage and petechiae, together with ecchymoses and hair shaft abnormalities, closely resembled palpable purpura, raising strong suspicion for small vessel vasculitis.

Given this constellation of findings, including cutaneous manifestations, arthralgia, and cytopenias, initial differentials were directed towards IgA vasculitis (Henoch–Schönlein purpura), cryoglobulinemia, autoimmune disorders, haematological malignancy, and chronic infections, such as hepatitis C, Epstein–Barr virus, and HIV. The patient was therefore treated empirically with corticosteroids, with minimal clinical response.

This case highlights the pseudovasculitic nature of scurvy, in which clinical features mimic small vessel vasculitis despite the absence of true vascular inflammation. Unlike vasculitis, which is characterised histologically by leukocytoclastic inflammation, nuclear debris and fibrinoid necrosis of vessel walls [5], scurvy results from impaired collagen synthesis leading to capillary fragility and red blood cell extravasation without inflammatory infiltrate. In our patient, the absence of systemic involvement, negative autoimmune serology, normal liver and renal functions and non-inflammatory findings on skin punch biopsy were key features in distinguishing scurvy from vasculitis. This distinction is crucial, as misdiagnosis may result in unnecessary immunosuppressive therapy, as initially occurred in this case.

On re-evaluation, a detailed dietary history revealed a markedly restrictive diet consisting predominantly of iced coffee for approximately three years. This prompted the consideration of vitamin C deficiency despite its rarity in high-income settings. Biochemical confirmation of severe vitamin C deficiency, together with rapid clinical improvement following supplementation, supported the diagnosis of scurvy.

Scurvy affects multiple organ systems and presents with a wide range of clinical manifestations. Dermatological features include perifollicular haemorrhage, petechiae, ecchymoses and coiled hairs, as observed in our patient [2]. Musculoskeletal involvement is common, with patients experiencing myalgia, bone pain, and arthralgia, typically affecting the knees and ankles [2]. Systemic manifestations include fatigue, malaise, anorexia, low mood and depression, some of which were observed in our patient [6].

Anaemia is a common finding in scurvy and is often multifactorial. Contributing factors include coexisting micronutrient deficiencies, particularly iron, folate and vitamin B12. This is explained by the role of vitamin C in enhancing intestinal iron absorption and in the conversion of folate to its active form, both of which are essential for erythropoiesis [7]. In addition, capillary fragility may lead to occult blood loss. In our patient, laboratory findings were consistent with iron deficiency anaemia in the context of severe nutritional deficiency.

Imaging findings in scurvy are variable and may not always be present [8]. However, they can provide supportive evidence. In our case, magnetic resonance imaging demonstrated soft tissue oedema and fluid along the flexor hallucis longus tendon sheath. These findings may represent underlying soft tissue or perimuscular haemorrhage secondary to capillary fragility. Similar imaging appearances have been reported, including cases of spontaneous intramuscular haemorrhage as a presenting feature of scurvy [9]. In this context, the imaging findings likely reflect early haemorrhagic changes rather than an inflammatory or infective aetiology.

## 4. Conclusions

This case highlights scurvy as a rare but important differential diagnosis in patients presenting with vasculitis-like dermatoses. It emphasises the importance of a detailed dietary history and identification of risk factors, particularly in individuals with restrictive eating patterns or chronic gastrointestinal symptoms. Recognition of characteristic dermatological features is essential, even in high-income settings. Given its nonspecific presentation, scurvy requires a high index of clinical suspicion. Early diagnosis enables prompt treatment, resulting in rapid symptom improvement and reduced morbidity.

## Figures and Tables

**Table 1:** Summary of laboratory investigations on presentation.

Observation	Results	Reference Range
Haemoglobin	63 g/L	115–155
MCV	61 fL	80–99
MCH	21.3 pg	26–34
Iron	2.8 μmol/L	9.0–30.4
Ferritin	32 μg/L	5–204
Transferrin saturation	3%	15–45
White cell count	3.7 × 10^9^/L	4.0–12.0
Platelet count	144 × 10^9^/L	150–400
Sodium	131 mmol/L	135–145
Potassium	3.2 mmol/L	3.5–4.5
Magnesium	0.6 mmol/L	0.7–1.1
Calcium	2.07 mmol/L	2.1–2.6
Vitamin B12	637 pmol/L	138–652
Vitamin C	<3 μmol/L	23–85
Folate	5 nmol/L	7.0–46.0

## Data Availability

The original contributions presented in this study are included in the article. Further inquiries can be directed to the corresponding author.
